# Supplementary material for: Prevalence of Obesity and Associated Risk Factors among Children and Adolescents in the Eastern Cape Province
Source: Int J Environ Res Public Health. 2022 Mar 3;19(5):2946. doi: 10.3390/ijerph19052946 (PMC8910414; doi:10.3390/ijerph19052946)
Supplement: Supplementary file 1 [file ijerph-19-02946-s001.zip › ijerph-1564997-supplementary.pdf]

SUPPLEMENTARY DATA

Table S1. Nutritional value of foods eaten during breakfast.

**Age Groups**	**Males**				**Females**			
	**9 - 13**		**14 - 18**		**9 - 13**		**14 - 18**	
	**RDA**		**RDA**		**RDA**		**RDA**	
N	39		38	16	58		46	12
Energy (kj)	2279	314.59	3152	322.87	2071	211.53	2368	266.72
Protein (g)	34	1.54	52	1.58	34	1.03	46	1.30
Protein %E		8.32		8.32		8.28		8.29
Plant protein (g)		1.39		1.43		0.93		1.18
Animal protein (g)		0.14		0.14		0.09		0.12
Fat (g)		3.5		3.62		2.37		2.99
Fat %E		42.28		42.61		42.58		42.60
Saturated fat (g)		0.69		0.71		0.47		0.59
Saturated fat %E		8.33		8.36		8.44		8.41
MUFA (g)	12	1.49	16	1.53	10	1	10	1.26
MUFA %E		18.00		18.01		17.96		17.95
PUFA (g)	1.2	1.15	1.6	1.18	1.0	0.77	1.1	0.97
PUFA %E		13.89		13.89		13.83		13.82
Cholesterol (mg)		0.28		0.29		0.19		0.24
Carbohydrate (g)	130	8.59	130	8.82	130	5.78	130	7.28
Carbohydrate %E		46.42		46.44		46.45		46.40
Fibre (g)	31	0.70	38	0.73	26	0.48	26	0.6
Added sugar (g)		2.97		3.05		2		2.52
Sugar %E		16.05		16.06		16.07		16.06
Added salt		0.06		0.06		0.04		0.05
**Age Groups**	**Males**				**Females**			
	**9 – 13**		**14 - 18**		**9 – 13**		**14 - 18**	
	**RDA**		**RDA**		**RDA**		**RDA**	
N	39		38	16	58		46	12
Calcium (mg)	1300	12.31	1300	12.31	1300	8.28	1300	10.43
Iron (mg)	8	0.49	11	0.50	8	0.33	15	0.42
Zinc (mg)	8	0.096	11	0.099	8	0.065	9	0.082
Vitamin A (μg)	600	4.69	900	4.82	600	3.16	700	3.98
Thiamin (mg)	0.9	0.057	1.2	0.058	0.9	0.038	1.0	0.048
Riboflavin (mg)	0.9	0.081	1.3	0.083	0.9	0.055	1.0	0.069
Niacin (mg)	12	0.73	16	0.75	12	0.49	14	0.62
Vitamin B6 (mg)	1.0	0.055	1.3	0.056	1.0	0.037	1.2	0.046
Folate (μg)	300	9	400	9.2	300	6.05	400	7.63
Vitamin B12 (μg)	1.8	0.087	2.4	0.089	1.8	0.059	2.4	0.074
Vitamin C (mg)	45	0.31	75	0.32	45	0.21	65	0.26
Vitamin D (μg)	15	0.086	15	0.088	15	0.058	15	0.073
Vitamin E (mg)	11	0.05	15	0.051	11	0.034	15	0.042

Table S2. Nutrient adequacy ratio by gender for breakfast.

Age Groups	Males				Females			
	9 - 13		14 - 18		9 – 13		14 - 18	
	RDA		RDA		RDA		RDA	
N	39		38	16	58		46	12
NAR Iron (%)	8	6.1	11	4.5	8	4.1	15	2.8
NAR Zinc (%)	8	1.2	11	0.9	8	0.8	9	0.9
NAR Vitamin A (%)	600	0.82	900	0.5	600	0.5	700	0.6
NAR Thiamin (%)	0.9	6.3	1.2	4.8	0.9	4.2	1.0	4.8
NAR Riboflavin (%)	0.9	9	1.3	6.4	0.9	6.1	1.0	6.9
NAR Niacin (%)	12	6.1	16	4.7	12	4.1	14	4.4
NAR Vitamin B6 (%)	1.0	5.5	1.3	4.3	1.0	3.7	1.2	3.8
NAR Folate (%)	300	3	400	2.3	300	2	400	1.9
NAR Vitamin B12 (%)	1.8	4.8	2.4	3.7	1.8	3.3	2.4	3.1
NAR Vitamin C (%)	45	0.7	75	0.4	45	0.5	65	0.4
MAR		4.4		3.25		2.9		3

*NAR: Nutrient adequacy ratio; MAR: Mean adequacy ratio

Table S3. Nutritional value for foods eaten during lunch served at school.

Age Groups	Males				Females			
	9 - 13		14 - 18		9 - 13		14 – 18	
	RDA		RDA		RDA		RDA	
N	39		38	16	58		46	12
Energy (kj)	2279	355.44	3152	364.79	2071	239	2368	301.35
Protein (g)	34	4.23	52	4.34	34	2.84	46	3.59
Protein %E		20.23		20.23		20.20		20.25
Plant protein (g)		2.32		2.39		1.57		1.98
Animal protein (g)		1.9		1.95		1.28		1.61
Fat (g)		2.95		3.03		1.98		2.5
Fat %E		31.65		31.56		31.48		31.52
Saturated fat (g)		0.79		0.81		0.53		0.67
Saturated fat %E		8.45		8.44		8.43		8.45
MUFA (g)	12	0.99	16	1.02	10	0.67	11	0.84
MUFA %E		10.58		10.63		10.65		10.59
PUFA (g)	1.2	0.94	1.6	0.97	1.0	0.63	1.1	0.8
PUFA %E		10.05		10.10		10.02		10.09
Diet P/S ratio								
Cholesterol (mg)		16.23		16.66		10.91		13.76
Carbohydrate (g)	130	9.03	130	9.27	130	6.07	130	7.66
Carbohydrate %E		43.19		43.20		43.18		43.21
Fibre (g)	31	1.56	38	1.6	26	1.05	26	1.32
Added sugar (g)		0.63		0.64		0.42		0.53
Sugar %E		3.01		2.98		3.00		3.00
Added salt		0.37		0.38		0.25		0.31
Salt %								
Age Groups	Males				Females			

	9 - 13		14 - 18		9 – 13		14 - 18	
	**RDA**		**RDA**		**RDA**		**RDA**	
N	39		38	16	58		46	12
Calcium (mg)	1300	28.62	1300	29.37	1300	19.24	1300	24.27
Iron (mg)	8	0.77	11	0.8	8	0.52	15	0.66
Zinc (mg)	8	0.63	11	0.65	8	0.43	9	0.54
Vitamin A (μg)	600	105.77	900	108.55	600	71.12	700	89.67
Thiamin (mg)	0.9	0.07	1.2	0.08	0.9	0.05	1.0	0.06
Riboflavin (mg)	0.9	0.06	1.3	0.06	0.9	0.04	1.0	0.05
Niacin (mg)	12	1.09	16	1.12	12	0.74	14	0.92
Vitamin B6 (mg)	1.0	0.16	1.3	0.16	1.0	0.11	1.2	0.13
Folate (μg)	300	23.17	400	23.79	300	15.59	400	19.65
Vitamin B12 (μg)	1.8	0.4	2.4	0.41	1.8	0.27	2.4	0.34
Vitamin C (mg)	45	7.6	75	7.84	45	5.14	65	6.48
Vitamin D (μg)	15	0.42	15	0.43	15	0.258	15	0.36
Vitamin E (mg)	11	0.7	15	0.71	11	0.47	15	0.59

Table S4. Nutrient adequacy ratio by gender for foods eaten during lunch served at school.

**Age Groups**	**Males**				**Females**			
	**9 - 13**		**14 - 18**		**9 – 13**		**14 - 18**	
	**RDA**		**RDA**		**RDA**		**RDA**	
N	39		38	16	58		46	12
NAR Iron (%)	8	9.6	11	7.3	8	6.5	15	4.4
NAR Zinc (%)	8	7.9	11	5.9	8	5.4	9	6
NAR Vitamin A (%)	600	17.6	900	12.1	600	11.9	700	12.8
NAR Thiamin (%)	0.9	7.8	1.2	6.7	0.9	5.6	1.0	6
NAR Riboflavin (%)	0.9	6.7	1.3	4.6	0.9	4.4	1.0	5
NAR Niacin (%)	12	9.1	16	7	12	6.2	14	6.6
NAR Vitamin B6 (%)	1.0	16	1.3	12.3	1.0	11	1.2	10.8
NAR Folate (%)	300	7.7	400	5.9	300	5.2	400	4.9
NAR Vitamin B12 (%)	1.8	22.2	2.4	17.1	1.8	15	2.4	14.2
NAR Vitamin C (%)	45	16.9	75	10.45	45	11.4	65	10
MAR		12.2		8.9		8.3		8.1

*NAR: Nutrient adequacy ratio; MAR: Mean adequacy ratio

Table S5. Nutrient adequacy ratio for foods eaten at home after school and during dinner.

Age Groups	Males				Females			
	**9 – 13**		**14 – 18**		**9 – 13**		**14 – 18**	
	**RDA**		**RDA**		**RDA**		**RDA**	
N	39		38	16	58		46	12
NAR Iron (%)	8	9.5	11	7.1	8	6.4	15	4.3
NAR Zinc (%)	8	7.5	11	5.6	8	5.1	9	5.7
NAR Vitamin A (%)	600	17.4	900	11.9	600	11.7	700	12.7
NAR Thiamin (%)	0.9	7.8	1.2	5.8	0.9	5.6	1.0	6
NAR Riboflavin (%)	0.9	5.6	1.3	3.8	0.9	4.4	1.0	4
NAR Niacin (%)	12	9	16	6.9	12	6.1	14	6.6
NAR Vitamin B6 (%)	1.0	16	1.3	13.1	1.0	11	1.2	11.7
NAR Folate (%)	300	7.6	400	5.8	300	5.1	400	4.8
NAR Vitamin B12 (%)	1.8	21.7	2.4	16.7	1.8	14.4	2.4	13.8
NAR Vitamin C (%)	45	5.6	75	3.5	45	3.8	65	3.3
MAR		10.8		9.7		7.4		8.5

*NAR: Nutrient adequacy ratio; MAR: Mean adequacy ratio

Table S6. Nutritional value of foods eaten after school and during dinner.

Age Groups	Males				Females			
	**9 - 13**		**14 – 18**		**9 - 13**		**14 - 18**	
	**RDA**		**RDA**		**RDA**		**RDA**	
N	39		38	16	58		46	12
Energy (kj)	2279	338.21	3152	347.11	2071	227.41	2368	286.74
Protein (g)	34	3.85	52	3.95	34	2.59	46	3.27
Protein %E		19.35		19.35		19.14		19.39
Plant protein (g)		2.3		2.36		1.55		1.95
Animal protein (g)		1.85		1.89		1.24		1.57
Fat (g)	25-35	2.92	25-35	3	25-35	1.97	25-35	2.48
Fat %E		32.81		32.84		32.92		32.87
Saturated fat (g)		0.79		0.81		0.53		0.67
Saturated fat %E		8.88		8.87		8.86		8.88
MUFA (g)	12	0.99	16	1.01	10	0.66	11	0.83
MUFA %E		11.12		11.05		10.03		11.00
PUFA (g)	1.2	1.85	1.6	1.90	1.0	1.25	1.1	1.57
PUFA %E		20.79		20.80		20.89		20.81
Cholesterol (mg)		16.21		16.63		10.9		13.74
Carbohydrate (g)	130	16.98	130	17.43	130	11.42	130	14.4
Carbohydrate %E		85.35		85.36		85.37		85.37
Fibre (g)	31	1.42	38	1.46	26	0.96	26	1.21
Added sugar (g)		0.74		0.76		0.5		0.63
Sugar %E		3.72		3.72		3.74		3.74
Added salt		0.36		0.37		0.25		0.31

Table S7. Nutritional value of foods eaten after school and during dinner.

Age Groups	Males				Females			
	9 - 13		14 – 18		9 – 13		14 – 18	
	RDA		RDA		RDA		RDA	
N	39		38	16	58		46	12
Calcium (mg)	1300	26.52	1300	27.22	1300	17.83	1300	22.49
Iron (mg)	8	0.76	11	0.78	8	0.51	15	0.65
Zinc (mg)	8	0.60	11	0.62	8	0.41	9	0.51
Vitamin A (μg)	600	104.49	900	107.24	600	70.26	700	88.59
Thiamin (mg)	0.9	0.07	1.2	0.07	0.9	0.05	1.0	0.06
Riboflavin (mg)	0.9	0.05	1.3	0.05	0.9	0.04	1.0	0.04
Niacin (mg)	12	1.08	16	1.11	12	0.73	14	0.92
Vitamin B6 (mg)	1.0	0.16	1.3	0.17	1.0	0.11	1.2	0.14
Folate (μg)	300	22.74	400	23.34	300	15.29	400	19.28
Vitamin B12 (μg)	1.8	0.39	2.4	0.40	1.8	0.26	2.4	0.33
Vitamin C (mg)	45	2.54	75	2.61	45	1.71	65	2.15
Vitamin D (μg)	15	0.41	15	0.42	15	0.28	15	0.35
Vitamin E (mg)	11	0.69	15	0.71	11	0.47	15	0.59
